# Effect of Brazing Temperature and Holding Time on the Interfacial Microstructure and Properties of TC4-Brazed Joints with Ti-Zr-Cu-Ni Amorphous Filler

**DOI:** 10.3390/ma18112471

**Published:** 2025-05-24

**Authors:** Yibin Wu, Jie Li, Zexin Wang, Sheng Lu, Kun Liu

**Affiliations:** School of Materials Science and Engineering, Jiangsu University of Science and Technology, Zhenjiang 212100, China; 13993148987@163.com (Y.W.); lijie081790@hotmail.com (J.L.); wangzexin@just.edu.cn (Z.W.); lusheng_ktz@just.edu.cn (S.L.)

**Keywords:** vacuum brazing, microstructure, Ti-Zr-Cu-Ni amorphous filler, TC4

## Abstract

A TC4 alloy was joined with Ti-Zr-Cu-Ni amorphous filler by vacuum brazing. The paper further explored how different brazing temperatures with a 20 min holding time, or varying holding times at a brazing temperature of 900 °C, impact the interface width, microstructure, composition distribution, microhardness, shear strength, and fracture surface of the brazed joints. The findings indicated that as the brazing temperature increased, the interface width became wider. Moreover, as the brazing temperature continued to rise, both the size of the Widmanstätten structure and the amount of the (Ti, Zr)_2_(Cu, Ni) brittle phase increased continuously, leading to the joint exhibiting harder and more brittle properties. As the temperature rose from 860 °C to 900 °C, the microhardness went up from 462.8 HV_0.1_ to 482.6 HV_0.1_. But when the temperature continued to increase (920 °C, 940 °C), the microhardness started to decrease, until it reached 392.6 HV_0.1_ at a holding time of 20 min. As the brazing temperature increased, the width of the joint interface expanded, and the shear strength continued to rise. When the brazing temperature rose to 940 °C, the shear strength increased to 223.9 MPa under a holding time of 20 min. With the prolongation of the holding time (from 10 min to 30 min), the Widmanstätten structure at the joint interface continuously grew towards the center. Additionally, the (Ti, Zr)_2_(Cu, Ni) phase and eutectic structure were separated by the Widmanstätten structure. The microhardness and shear strength reached their maximum values at 900 °C, and the shear strength was measured at 137.6 MPa.

## 1. Introduction

Titanium alloys possess characteristics such as high heat resistance, high corrosion resistance, high specific strength, and light weight [1]. They are often applied in fields such as aerospace, railway transportation, medical treatment, and biology [2,3,4,5]. Ti-6Al-4V has two allotropes: α-Ti, which has an HCP structure and coexists with β-Ti in the α+β two-phase region below its β transus temperature (approximately 995 °C), and β-Ti, which has a BCC structure and becomes the dominant phase above 995 °C [6,7]. TC4 is an abbreviation for Ti-6Al-4V. In the following text, TC4 will be used instead of Ti-6Al-4V. During the fusion welding process of manufacturing TC4 alloy parts with complex structures, a substantial number of intermetallic compounds (IMCs) are generated due to the high temperature. This causes the parts to become more brittle and makes them have a high crack susceptibility. Moreover, titanium alloys have high chemical reactivity and are prone to combining with carbon, hydrogen, and oxygen in the air, which is detrimental to the properties of materials. To avoid these problems, vacuum brazing with a relatively low brazing temperature is generally adopted in the joining of titanium alloys [8,9].

Titanium-based amorphous filler metals have the advantages of uniform composition, high assembly precision, excellent welding strength, corrosion resistance, and good wetting and spreading properties [10,11]. These advantages make them suitable to be used as a brazing filler material for TC4. The IMCs form by the transformation of the crystal structure of titanium alloys during cooling, which reduces the properties of brazing joints. For the purpose of suppressing the formation of IMCs, vacuum brazing with amorphous filler is usually employed to minimize residual stress and deformation and enhance the mechanical properties of brazed joints [12]. According to previous papers, Ti-Zr-Cu-Ni alloys are commonly utilized as amorphous filler materials for the vacuum brazing of TC4 [13,14,15]. This filler material features a low melting point range and typically does not degrade the properties of the brazed joint [16,17]. As the most important parameters of this process, the temperature applied during brazing and the duration of holding time frequently become subjects of investigation for numerous scholars [18,19,20]. For example, with the purpose of enhancing the properties of the Zircaloy-4, which is a fuel cladding material for light water reactors, Yang et al. [13] carried out vacuum brazing of Zircaloy-4/Ti_3_AlC_2_ using the Ti-Zr-Cu-Ni amorphous filler metal. This study demonstrated that a peak shear stress of 187 MPa is achieved at a brazing temperature of 940 °C with a 5 min holding time. Moreover, this study also explained the mechanism of the influence of Zr-based alloy particles on joint strength. Furthermore, Cai et al. [21] employed Ti-Zr-Cu-Ni amorphous filler metal to braze γ-TiAl alloy. With the growing brazing temperature, the proportions of α-Ti phase and α_2_ phase increased, whereas the content of IMCs declined. As the brazing temperature reached the target value of 930 °C, the ultimate tensile strength reached its maximum value of 468 MPa.

According to the above-mentioned research, it would be scientifically significant to carry out vacuum brazing on titanium alloys utilizing Ti-Zr-Cu-Ni amorphous filler metal by regulating the holding time and brazing temperature. Jing et al. [22] selected three kinds of Ti-Zr-Cu-Ni filler metals with different Zr contents to investigate the influence of the composition of Ti-Zr-Cu-Ni filler on the brazing of TC4 alloy. They found that, owing to the solid solution strengthening effect, increasing the content of Zr in the filler metal can effectively enhance the strength and hardness of the joint. Additionally, this work exhibited that the joint properties of the 18Zr filler metal were the best. However, this article did not research the influence of the brazing temperature and the holding time on the joint. For this reason, Ling et al. [12] investigated the microstructural evolution and elemental diffusion behavior in the course of vacuum brazing at 940 °C. Under a holding time condition of 90 min, the tensile strength reaches a maximum value (984.9 MPa), and the elongation is 12.39%. The experiment indicated that both brazing temperature and holding time impacted elemental diffusion capacity, thus changing the transformation temperature of β-Ti and influencing the content of the eutectic structure. However, the parameter research scope of this paper is not comprehensive enough. Additionally, the influence on the shear properties of the material has not been mentioned either. Compared with their studies, this work discusses the effects of different brazing temperatures or holding times, within a wider parameter range, on the brazed joints and their shear strength.

In this study, Ti-Zr-Cu-Ni amorphous foils is used to braze TC4 alloy by vacuum brazing. The selection of filler metal during the brazing process is important to the formation of joints. Amorphous foils have excellent wettability or structural properties compared to ordinary metal foils. The manufacturing process of amorphous foil is usually achieved through rapid cooling, which can save processing steps and reduce production costs compared with ordinary metal materials. Additionally, in this work, the composition of Ti-Zr-Cu-Ni filler amorphous foil is different from that used in previous work. The composition of filler foil has an effect on the selection of processing parameters (such as brazing temperature and holding time). This paper aims to put forward the mechanism of influence of brazing parameters on joint properties and to point out the optimization scheme for brazing parameters. The effect of brazing temperature and holding time on the brazed joint with this new filler amorphous foil will be revealed in our work, which can provide guidance for selecting optional process parameters in industrial practice.

## 2. Materials and Methods

In this experiment, TC4 alloy served as the base metal for brazing, and its main alloying elements are Ti, Al, and V elements. The base metals are all rectangular metal blocks with dimensions of 15 mm × 15 mm × 4 mm. The brazing filler material used in this experiment is an amorphous foil with a composition of Ti-Zr-Cu-Ni. The foil thickness is approximately 30 μm, and the width is about 30 mm. The amorphous brazing foil was fabricated with reference to the research conducted by E. Ganjeh et al. [23]. Their research findings indicated that when the rapid solidification technique is applied to fabricate the Ti-Zr-based amorphous brazing foil, it can enhance the brazing fluidity and wettability, and make the microstructure distribution of the brazed joint more uniform. The chemical compositions of the base metal and the amorphous foil are listed in Table 1.

**Figure 1:**
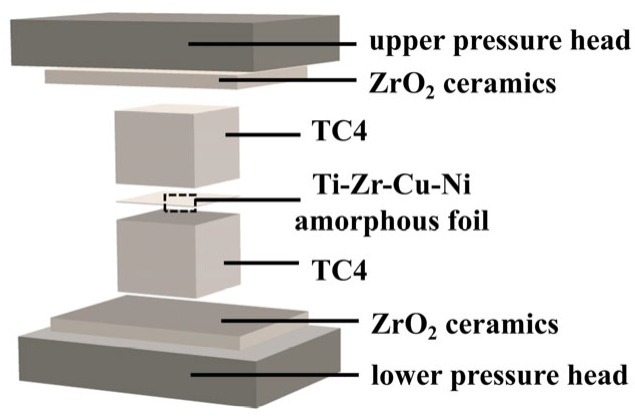
Schematic diagram of assembly sequence during TC4 vacuum-brazing process.

**Figure 2:**
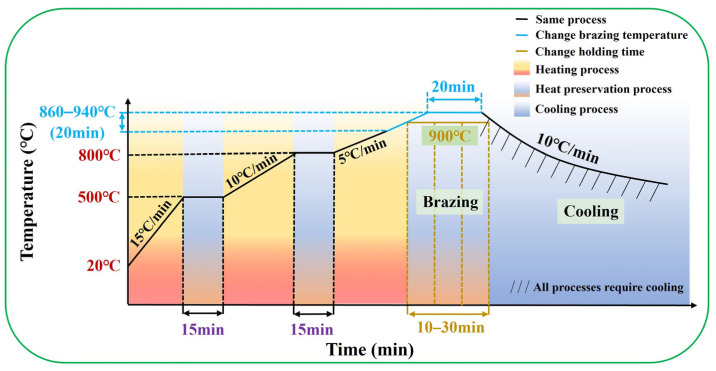
The heating process curves of TC4 brazing specimens under different brazing temperatures and different holding times.

A vacuum resistance heating furnace (WZB-20 type) (Zhongshan Kaixuan Vacuum Science & Technology Co., Ltd., Zhongshan, China) was used for brazing and the vacuum level was set as 10^−4^ Pa. To investigate the effects of brazing temperature and holding time on the brazed base metal, this experiment controlled the same brazing temperature and observed the specimens with different holding times. Similarly, the experiment also controlled the same holding time and observed the specimens at different brazing temperatures. The brazing temperature was regulated within a range of 860 °C–940 °C, increasing successively at intervals of 20 °C for each group of tests. During this holding phase, the holding time was maintained at 20 min. At holding times of 10, 20, and 30 min, the brazing temperature is controlled to remain stable at 900 °C. The cooling rate is 10 °C/min. Finally, seven groups of brazed specimens can be obtained. The brazing process parameters for TC4 alloy and the specimen numbers of TC4 alloy are listed in Table 2. In the heating-up stage, the TC4 alloy brazed specimens mainly rely on a three-stage segmented heating method to gradually increase the temperature until the pre-set brazing temperature is reached. The schematic diagram of the process assembly sequence for TC4 alloy is shown in Figure 1. After reaching the specified brazing temperature, it will enter the holding stage. At this time, the holding time represents a critical part of the brazing process parameters for TC4 alloy. When the holding stage is completed, it will enter the cooling process. Figure 2 illustrates the temperature variation curve over time during the brazing process of TC4 alloy specimens.

**Figure 3:**
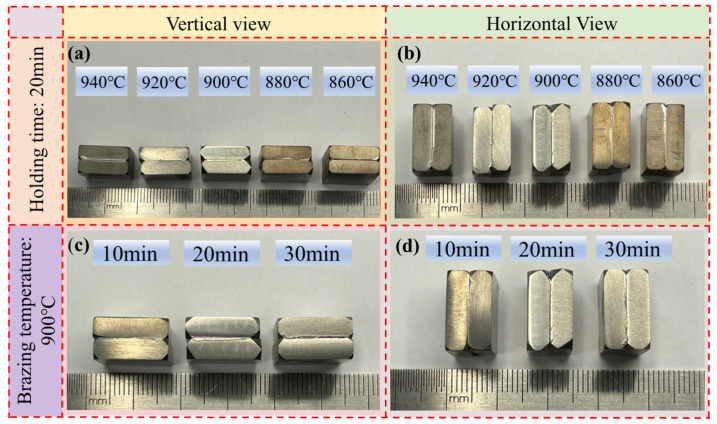
Macroscopic morphology of the sample: (**a**) vertical view samples at 20 min, (**b**) horizontal view sample at 20 min, (**c**) vertical view sample under 900 °C, (**d**) vertical view sample under 900 °C.

After brazing, the metallographic specimens of the TC4 titanium alloy-brazed joints were prepared. The macroscopic morphology of the specimens is shown in Figure 3. After the specimens were cut, they were then embedded. The embedded specimens were polished with sandpaper. Subsequently, the specimens were polished with polishing paste on a metallographic polishing machine. Then, the specimens were etched with an etching solution consisting of 1mL of hydrofluoric acid, 2 mL of nitric acid, and 50 mL of water for 44–47 s. The surface of the brazed joint was rinsed with water and then cleaned with an ethanol solution. After the metallographic specimens were obtained, an optical microscope (OM) and scanning electron microscope (SEM) were used to observe and analyze the metallography of the microstructure at the brazed joint. The energy-dispersive X-ray spectroscopy (EDS) attached to the SEM was employed to conduct qualitative and quantitative elemental analysis of the brazed joint region. The SANS CMT520 universal testing machine (Shenzhen Suns Technology Stock Co., Ltd., Shenzhen, China) was used to test the shear strength of the specimens, and the loading speed was set at 0.5 mm/min. The shearing testing method has been presented in the previous work [24]. The semi-automatic Vickers hardness tester was employed to measure the microhardness at the brazed joint.

## 3. Results and Discussion

### 3.1. The Effect of Different Brazing Temperatures on Brazed Joint

**Figure 4:**
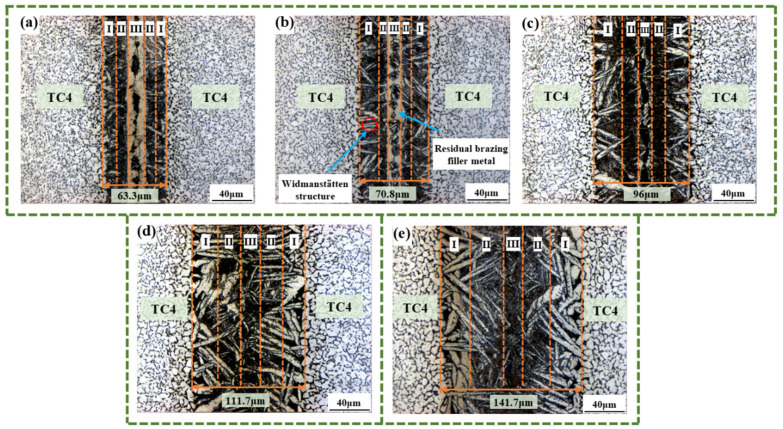
Microstructures of brazed joints at 20 min holding time under different brazing temperatures: (**a**) 860 °C, (**b**) 880 °C, (**c**) 900 °C, (**d**) 920 °C, and (**e**) 940 °C.

During the vacuum-brazing process, the TC4 alloy and the Ti-Zr-Cu-Ni amorphous brazing filler metal undergo mutual diffusion and reaction, creating a brazed joint zone at the central position of the TC4 base metal. For systematic investigation of the brazed joint’s microstructural features, the joint was partitioned into three distinct zones, namely the diffusion zone, the interface zone, and the joint central zone (corresponding to regions I, II, and III in Figure 4, respectively). The diffusion zone (Zone I) denotes the base metal regions flanking both sides of the brazed joint. The joint center zone (Zone III) is the area where the amorphous foil was located before brazing and is the region where mutual diffusion and reaction take place between the filler metal and the base metal during brazing. The interface zone (Zone II) lies between the diffusion zone and the joint center zone. The microstructure formed in this region is in a transitional state between the microstructures formed in Zone I and Zone III. Dividing this region facilitates the analysis of microstructural evolution in brazed joints under the effect of brazing parameters. Figure 4a illustrates the microstructure of the brazed joint of 1# (860 °C/20 min). From the figure, it can be observed that there is a very obvious white banded structure distributed in Zone III. These structures are the undiffused Ti-Zr-Cu-Ni amorphous brazing filler metal. The residual undiffused amorphous brazing filler metal in Zone III indicates that when the brazing temperature is below 880 °C, it is difficult to make this amorphous brazing filler metal diffuse completely. Observing Figure 4b, obvious parallel white acicular structures can be found in Zone I. Based on the microstructural evolution law of the TC4 alloy, these acicular structures are Widmanstätten structures, and the Widmanstätten structures originate at the TC4 base metal boundary and propagate toward the joint’s central zone. Figure 4c illustrates reciprocal diffusion between the Ti-Zr-Cu-Ni amorphous filler and TC4 alloys at 900 °C brazing temperature, contributing to effective brazing. From the observation of Zone I showed in Figure 4c–e, the Widmanstätten structure continues to coarsen, and the proportion of the Widmanstätten structure in Zone II is increasing. The Widmanstätten structure has poor plasticity and toughness, and its coarsening and extension should be avoided as much as possible.

**Figure 5:**
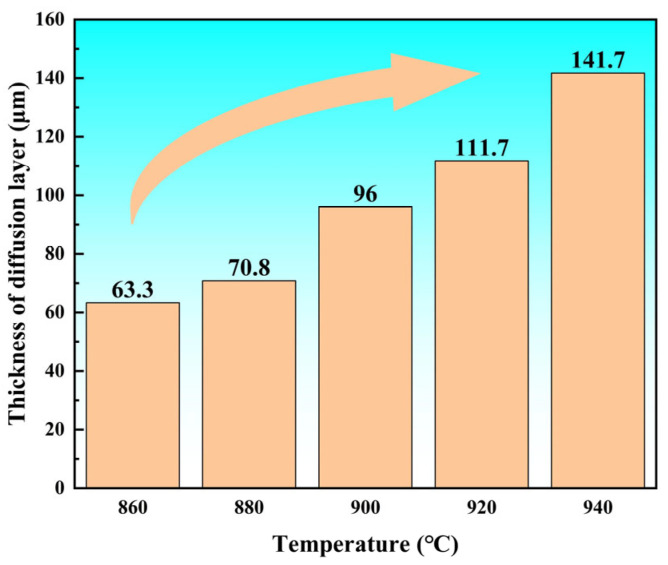
The width of the brazing interface at different brazing temperatures with the same holding time of 20 min.

After observing Figure 5, it can be found that the width of the joint interface is continuously increasing. V and Al in the TC4 alloy diffuse towards the Ti-Zr-Cu-Ni amorphous foil brazing filler metal, while Ni, Zr, and Cu in the Ti-Zr-Cu-Ni amorphous foil brazing filler metal diffuse towards the TC4 alloy. Hence, as the brazing temperature increases, the atomic thermal motion becomes more intense. Meanwhile, the diffusion activation energy decreases. As a result, the elements in the TC4 alloy and the Ti-Zr-Cu-Ni amorphous foil diffuse more vigorously, and the width of the brazed joint interface becomes larger.

In conclusion, to avoid incomplete diffusion of the brazing filler metal and excessive coarsening of the Widmanstätten structure, when the holding time is controlled at 20 min, it is advisable to choose 900 °C as the brazing temperature.

The atomic structure of Zr atoms is similar to that of Ti atoms, which enables the Zr element to achieve an infinite solid solution alongside α-Ti within the β-Ti matrix of TC4. The elements Zr and Ti can also form an intermetallic compound together with the elements Ni and Cu, namely (Ti, Zr)_2_(Cu, Ni), which exhibits hardness and brittleness [14]. Furthermore, the main phase compositions of the Ti-Zr-Cu-Ni brazing filler metal are α-(Ti, Zr) and (Ti, Zr)_2_(Cu, Ni).

**Figure 6:**
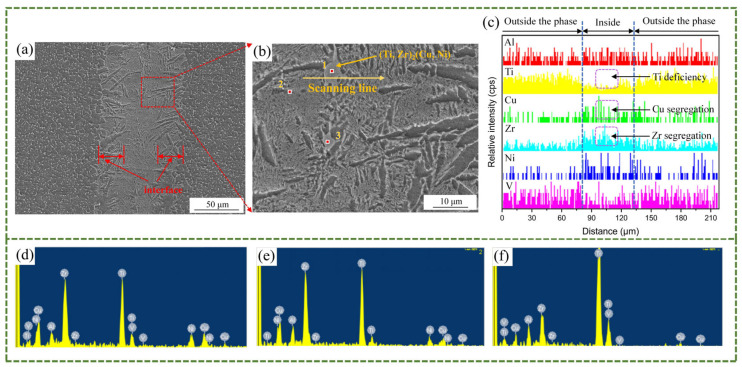
Microstructure and composition of the brazed joint interface at 900 °C: (**a**) microstructure of the interface, (**b**) the locally magnified microstructure of Figure (**a**), (**c**) the EDS results of the scanning line in Figure (**b**), (**d**) the EDS point scanning results of Point 1 in Figure (**b**), (**e**) the EDS point scanning results of Point 2 in Figure (**b**), (**f**) the EDS point scanning results of Point 3 in Figure (**b**).

**Figure 7:**
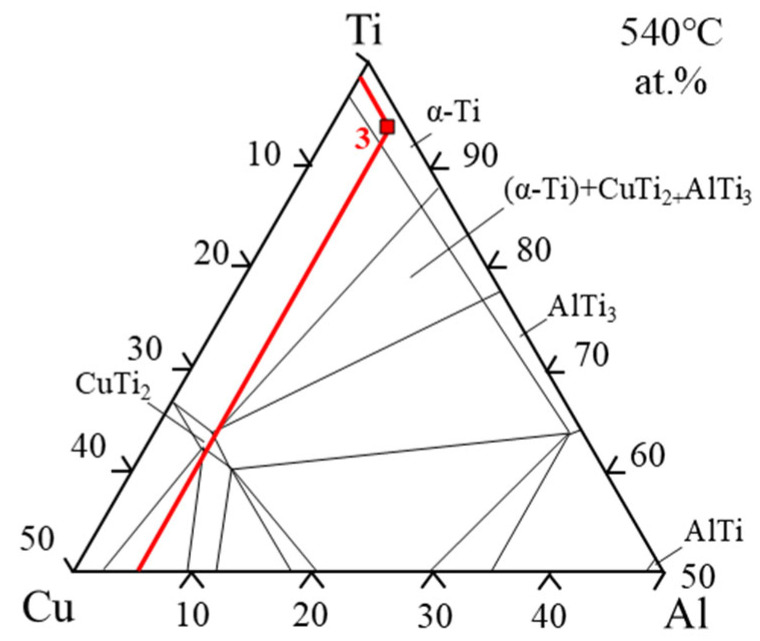
Ti-Cu-Al ternary phase diagram [26].

As mentioned above, when the process parameters are those of 3# (900 °C/20 min), it is likely to yield optimal property of the brazed joint. To investigate the microstructure and composition of this brazed joint, a composition study through linear scanning and point scanning by EDS was conducted on the TC4 alloy. Figure 6a clearly shows the transition zone at the brazed joint–base metal interface. These parallel dendrites which are located in the joint are the Widmanstätten structure. The joint transition area in Figure 6a was magnified as exhibited in Figure 6b. It is evident from the images that except for the Widmanstätten structure distributed on the right side of the joint transition zone, there are also grayish-white phases distributed in the transition zone. A line scan distribution analysis using EDS was carried out on the grayish-white phase to further analyze the composition. Figure 6c shows that the internal composition of Point 1 is a Ti-poor region and a Zr-enriched and Cu-enriched region. Table 3 shows the point scanning compositions corresponding to the three points marked in Figure 6b. The point scan images of the three points in Figure 6b correspond to Figure 6d–f, respectively. Point 1 is taken from the inside of the grayish-white microstructure. The ratio of Ti + Zr to Cu + Ni in the composition of this point is 1.8, and this value is close to 2. Therefore, it is speculated that the phase is (Ti, Zr)_2_(Cu, Ni). (Ti, Zr)_2_(Cu, Ni), as brittle IMCs should be minimized. Additionally, based on the analysis of the microscopic morphology at Point 2 and the results of point-scanning composition analysis, it can be known that this microstructure is a eutectic structure. As described in reference [25], it is a transient liquid phase bonding, which the brazing process can be regarded as. And in the liquid state of the brazing filler metal, it all transforms into the β-Ti precipitation phase. With the diffusion and solidification of the brazing filler metal, upon cooling to the eutectic temperature, β-Ti undergoes transformation into α-Ti. During this stage, with the continuous diffusion of Ni and Cu, they dissolve in β-Ti phase and keep migrating during the solidification process, thus obtaining a lamellar structure of (Ti, Zr)_2_(Cu, Ni) and α-Ti. The eutectic structure, which is located in the joint, is formed by this process. Moreover, based on the EDS point scanning analysis of Point 3, the Ti content is as high as 74.42 at%. With reference to the Ti-Cu-Al ternary phase diagram [26] and Figure 7, this phase is identified as a high-Ti aggregated α-Ti phase. This phase forms via the segregation of Ti from both the filler metal and base metal during their diffusion into the transition zone.

**Figure 8:**
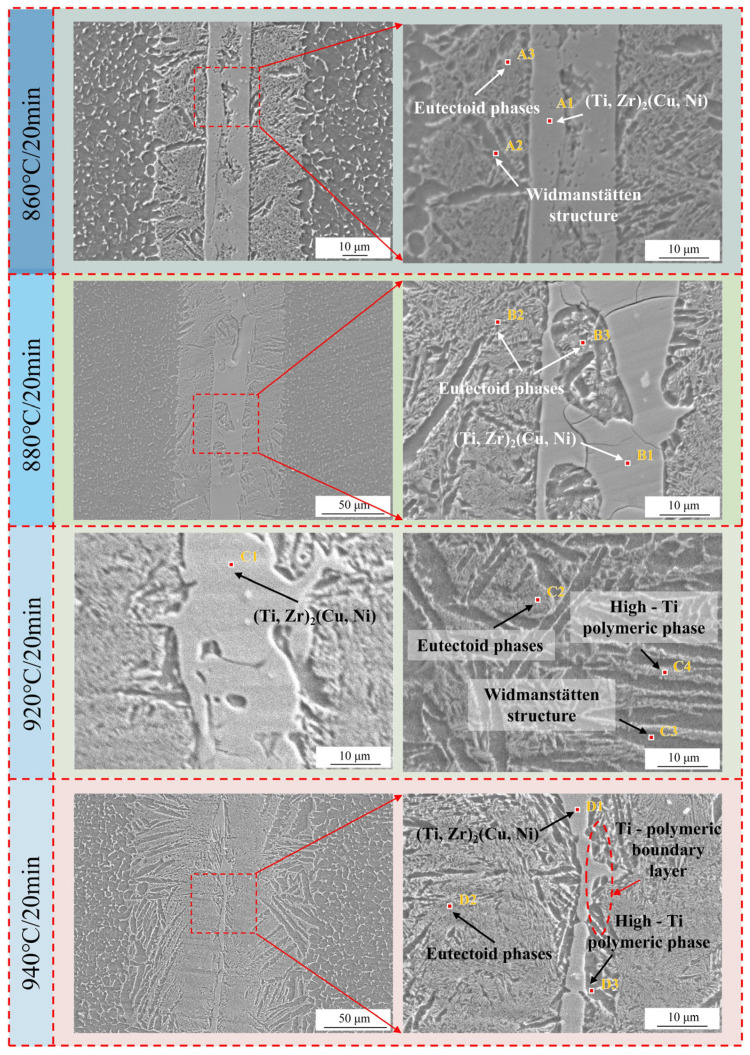
Microstructure and composition of the brazed joint interface at different temperatures.

To investigate the effect of brazing temperature on the microstructural composition of brazed joints, point scanning analyses were carried out on 1# (860 °C/20 min), 2# (880 °C/20 min), 3# (900 °C/20 min), 4# (920 °C/20 min), and 5# (940 °C/20 min), respectively, as shown in Figure 8. And 3# (900 °C/20 min) has already been shown in Figure 6. By combining the analysis of the microstructural diagram in Figure 8 and the corresponding EDS point scanning diagram in Table 4, it is evident that with the continuous increase in brazing temperature, the region in Zone I where (Ti, Zr)_2_(Cu, Ni) is enriched is continuously decreasing. This means that higher brazing temperatures accelerate the diffusion of Zr, Ni, and Cu into the TC4 alloy matrix, thereby reducing the formation areas of (Ti, Zr)_2_(Cu, Ni) in Zone I. Furthermore, after observing the structure of Zone II, it was found that (Ti, Zr)_2_(Cu, Ni), the eutectic structure, and α-Ti are all alternately distributed in this area. Based on the morphological features of the structure, it can be inferred that part of α-Ti is in the form of Widmanstätten structure, and part of it is the enrichment area of α-Ti. Moreover, influenced by the element concentration, the main phases and microstructures in different regions of the joint also vary. The primary phase distribution sequence from the base metal to the brazed joint is the Widmanstätten structure, eutectic structure, (Ti, Zr)_2_(Cu, Ni), and the enrichment area of α-Ti. Since Zr, Cu, and Ni elements continuously diffuse from the joint area towards the base metal, the contents of Zr, Cu, and Ni are lower the closer it is to the base metal. This results in the formation of a Ti-enriched layer adjacent to the base metal and the generation of a Widmanstätten structure. The Ti content at the interface between Zone II and Zone III is influenced by the diffusion of Zr, Ni, and Cu. In addition, according to the principle of solute exchange, the Ti content in Zone II and Zone III is low. Therefore, it is easier to form a lamellar eutectic structure and (Ti, Zr)_2_(Cu, Ni) in Zone II and Zone III.

**Figure 9:**
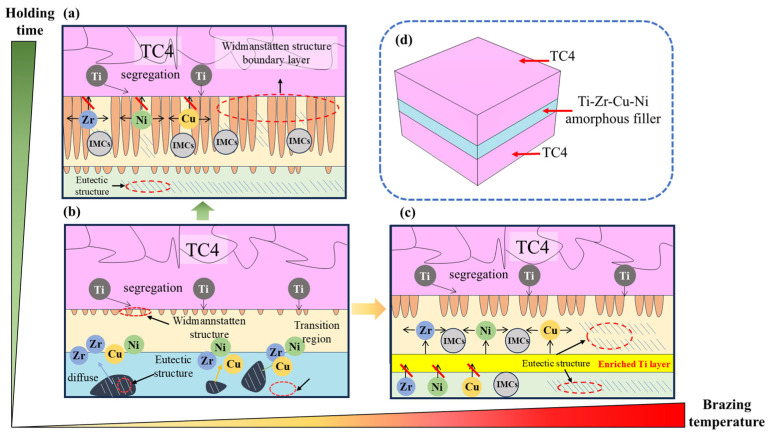
Influence mechanism of different brazing temperatures and different holding times on the vacuum brazed joints: (**a**) long holding time, (**b**) low brazing temperature and short holding time, (**c**) high brazing temperature, (**d**) schematic diagram of the specimen.

Figure 9b,c is a mechanism diagram of the effect of the brazing temperature on the brazed joint. And Figure 9d is a schematic diagram of the joint interface. In order to enable Ti-Zr-Cu-Ni amorphous film to flow fully within the brazed seam of base material, and thus fill the brazed seam, the brazing temperature for Ti-Zr-Cu-Ni filler metal is selected to be 25–100 °C above its solidus temperature. When the brazing temperature exceeds 900 °C, excessive interdiffusion occurs between the TC4 alloy base metal and Ti-Zr-Cu-Ni brazing filler metal. As a result, a violent reaction will occur, leading to the formation of additional brittle (Ti, Zr)_2_(Cu, Ni) intermetallic compounds. These IMCs will reduce the mechanical properties of the joint [27].

In the region adjacent to the base metal, the diffusion of Ti exerts a significant influence, and the Widmanstätten structure keeps growing. As brazing temperature increases, the diffusion of Zr, Cu, and Ni elements is accelerated, and the Widmanstätten structure is coarsened. The distribution of (Ti, Zr)_2_(Cu, Ni) and the eutectic structure at the joint interface are divided by the Widmanstätten structure and become more and more dispersed. Owing to the enrichment of Zr, Ni, and Cu elements in Zone II and Zone III, the diffusion rate of Ti is relatively low, and it does not have enough time to diffuse. Coupled with the relatively high Ti content, the undiffused Ti accumulates at the boundary of Zone III. When the temperature reaches 940 °C, an obvious Ti-rich boundary layer has been formed at Zone III’s boundary, as shown in Figure 8. This boundary layer will continuously widen with increasing brazing temperature, and this boundary layer significantly hinders Zr, Cu, and Ni diffusion at the joint interface. As a result, a portion of (Ti, Zr)_2_(Cu, Ni) remains at the joint after cooling, which may become the source of cracks [28,29].

To conclude, setting the brazing temperature to 900 °C can not only ensure sufficient diffusion of the brazing filler material, but also prevent the Widmanstätten structure from being excessively coarsened, thus avoiding a reduction in the property of the joint.

### 3.2. The Effect of Different Holding Times on Brazed Joints

**Figure 10:**
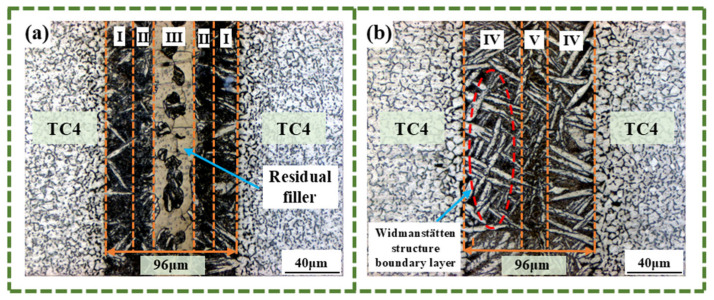
Microstructure of the brazed joint with a brazing temperature set at 900 °C and different holding times: (**a**) 10 min, (**b**) 20 min.

From the above analysis, it can be seen that it is more appropriate to keep the brazing temperature at 900 °C. Therefore, to investigate how varying holding times affect joint quality, the brazing temperature remained constant at 900 °C in this experiment, and the microstructures of the joints of Sample No. 3 (900 °C/20 min), Sample No. 6 (900 °C/10 min), and Sample No. 7 (900 °C/30 min) were observed, as illustrated in Figure 4c and Figure 10. In Figure 10a, at a holding time of 10 min (insufficient for full diffusion), a large amount of residual brazing filler metal remains undiffused from Zone I to Zone II and Zone III. In Figure 10b, the joint with a holding time of 30 min is divided into a Widmanstätten structure zone and a joint center zone (corresponding to Zone IV and Zone V in Figure 10b, respectively). Owing to the excessively long holding time of this process, the diffusion zone and the interface zone have completely merged, and a large number of acicular Widmanstätten structures are distributed within. Moreover, there are also some slender Widmanstätten structures in Zone V, but their sizes are relatively small and they have not yet grown. At a holding time of 30 min, it will cause the Widmanstätten structure to continuously grow from the base metal zone towards the center of the joint, and form a relatively thick boundary layer of the Widmanstätten structure. In addition, the width of the joint interface of the specimens under a brazing temperature of 900 °C with different holding times remains basically unchanged. This is because the extension of the holding time has no significant effect on the diffusion rate of each element. When Zr, Ni, and Cu diffuse from the joint’s center to the base material, they are hindered by the boundary layer formed by the continuous growth of the Widmanstätten structure. Moreover, the Widmanstätten structure is gradually divided into fine eutectic structures or IMCs by the coarsened Widmanstätten structure, and make the eutectic structure and IMCs evenly distributed on the matrix.

**Figure 11:**
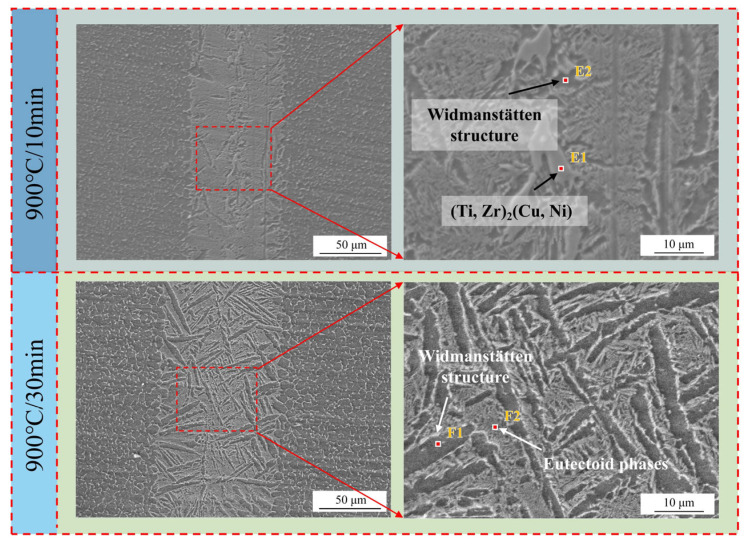
The microstructure and composition of the brazed joint interface under different holding times.

EDS point-scanning analysis was carried out on the Zone III and Zone V in Figure 10. Combining the components of the corresponding points in Figure 11 and Table 5, due to the diffusion of Zr, Cu, and Ni elements, the blocky structures present at the joint center are identified as eutectic structures and (Ti, Zr)_2_(Cu, Ni). Additionally, with the holding time extended to 30 min, the center of the joint interface is occupied by elongated Widmanstätten structures and eutectic structures cut by the Widmanstätten structures [30]. The eutectic structures exhibit discontinuous lamellar shapes. The presence of coarse Widmanstätten structures in Zones IV and V not only hinders element diffusion into the base metal, but also significantly weakens the material’s mechanical properties. The mechanism diagram of holding time’s effect on the brazed joint is presented in Figure 9a. As the holding time is prolonged, the Zr, Ni, and Cu in the brazing filler metal continuously diffuse into the base metal. Meanwhile, Ti elements in the base metal and brazing filler material continue to diffuse into the transition zone, causing the Widmanstätten structure to keep growing under the influence of heating. When heated to a certain extent, the elements in the brazing filler metal are separated by the boundary layer formed by the thick Widmanstätten structure at one end of the base metal. This makes the diffusion path of atoms through the grain boundaries more tortuous, preventing further diffusion and resulting in the width of the joint interface remaining unchanged. Apart from the fact that the Widmanstätten structure itself reduces the performance of the brazed joint interface, the Cu, Ni and Zr that cannot diffuse are separated by the Widmanstätten structure to generate brittle and hard (Ti, Zr)_2_(Cu, Ni), which further reduces the comprehensive performance of the joint.

### 3.3. Microhardness and Shear Strength of the Joint

**Figure 12:**
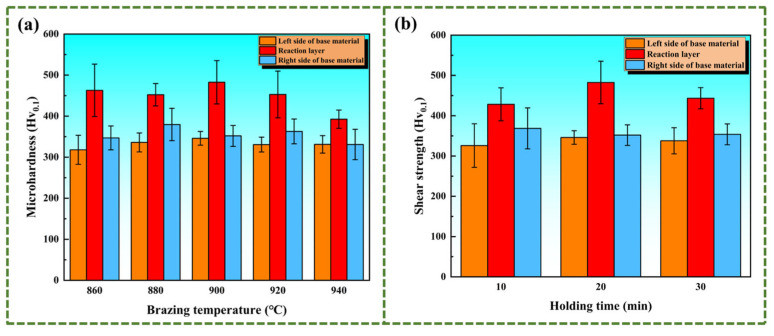
Microhardness profiles of brazing joints: (**a**) under different brazing temperature conditions with 20 min of holding time; (**b**) at 900 °C with different holding times.

The microhardness of the vacuum brazing specimens is presented in Figure 12. Figure 12a represents the microhardness results for specimens brazed at different temperatures. The microhardness of the reaction layer is significantly higher than that of the TC4 base metal regions on both sides, as shown in the figure. This is due to the solute strengthening effect brought about by the elements in the amorphous brazing filler metal. As the brazing temperature increases to 900 °C, the hardness of the central area of the brazed joint is the highest, reaching 482.6 Hv_0.1_. This indicates that after some elements of the amorphous brazing filler material have undergone sufficient diffusion, a uniform eutectic structure is formed, which enhances the strength of the joint. However, with the brazing temperature increasing further, due to the continuous growth of the Widmanstätten structure in the reaction zone and the aggregation of Ti elements at the boundary of Zone I, which hinders the continuous diffusion of elements, the strength of the joint begins to decline again. Figure 12b shows that the microhardness increases with longer holding times, likely due to improved interdiffusion at the brazed interface. Similarly, When the holding time is prolonged to 20 min, the elements in the amorphous brazing filler metal have diffused sufficiently, forming a certain amount of intermetallic compound (Ti, Zr)_2_(Cu, Ni), which plays a role in strengthening the joint. On the other hand, as the holding time continues to increase, the Widmanstätten structure coarsens, owing to the diffusion of Ti. As a result, a Widmanstätten structure boundary layer is formed, which reduces the interfacial strength at the brazed joint interface.

**Figure 13:**
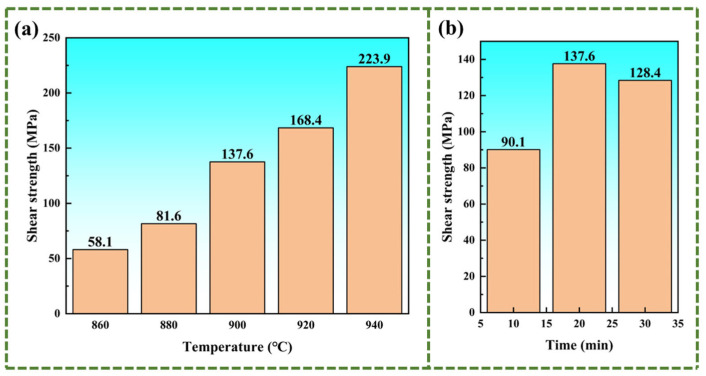
Shear strength of the brazing joints: (**a**) with a holding time of 20 min under different brazing temperatures; (**b**) at 900 °C with different holding times.

To measure the shear strength of the material, in order to explore the influence of brazing parameters, the specimens were subjected to a shear strength test. Figure 13 shows the shear strength of the specimens. The experiment results show that as the brazing temperature increases, the shear strength of the material continuously improves. As shown in Figure 13a, for the specimen brazed at 940 °C, its shear strength is close to 224 MPa. Because an increase in the brazing temperature can accelerate the diffusion and migration of elements. In particular, the elements Zr and Cu diffuse more rapidly, widening the width of the brazed joint interface and enabling the IMCs to be uniformly distributed in the transition zone. The IMCs significantly reduce the shear strength of the joint interface. Therefore, the increase in the width of the joint interface has a much greater impact on the shear strength than the growth of the Widmanstätten structure and the brittle and hard phases. Moreover, the shear strength of the samples at different holding times is exhibited in Figure 13b. Increasing the holding time does not augment the brazed joint interface width. As the holding time continues to be prolonged, only the negative impacts of the growth of the Widmanstätten structure and intermetallic compounds (IMCs) reduce the shear strength of the specimens. As a result, prolonging the holding time to 20 min is essential for achieving sufficient diffusion of filler metal elements. And the Widmanstätten structure and (Ti, Zr)_2_(Cu, Ni) have not fully grown. At this time, the shear strength reaches 137.6 MPa. If the holding time is further prolonged, the shear strength will instead decrease by Widmanstätten structure and (Ti, Zr)_2_(Cu, Ni).

**Figure 14:**
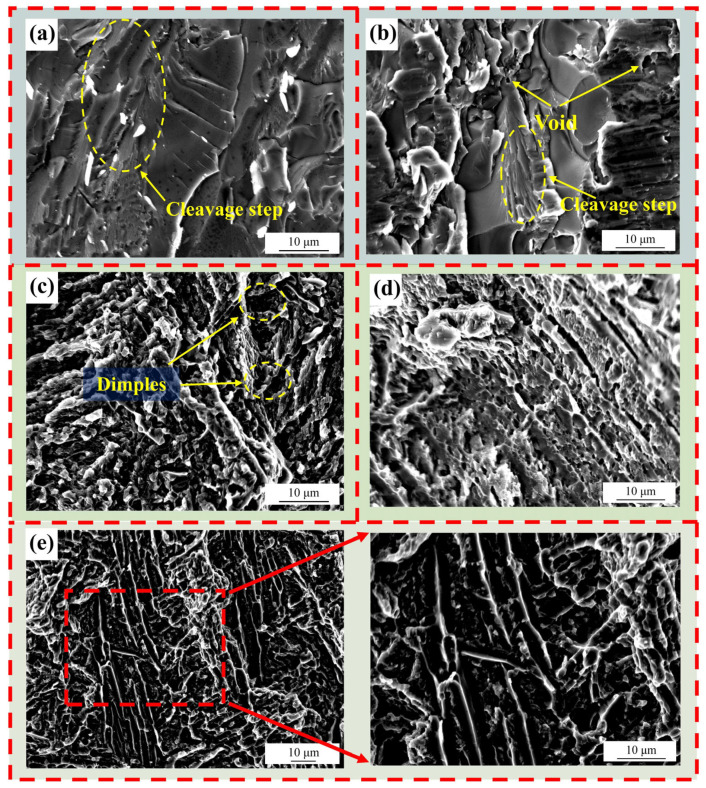
Fracture surface morphology at different brazing temperatures with holding time of 20 min: (**a**) fracture surface of joint at 860 °C, (**b**) fracture surface of joint at 880 °C, (**c**) fracture surface of joint at 900 °C, (**d**) fracture surface of joint at 920 °C, (**e**) fracture surface of joint at 940 °C.

The fracture morphologies from the shear test under different brazing temperatures are shown in Figure 14. As can be observed from Figure 14a, when the brazing temperature is 860 °C, there are obvious continuous cleavage steps at the fracture. Figure 14b shows the fracture morphology at 880 °C. At this time, not only are there parallel cleavage steps at the fracture, but there are also some pores and tear ridges. Both are typical intergranular fractures and exhibit the characteristics of brittle fractures. This indicates that their plasticity and toughness are not good. Cracks initiate and develop from the grain boundaries and finally lead to fracture. However, the appearance of pores and tear ridges indicates that the 2# (880 °C/20 min) has a tendency to transform towards ductile fracture. The reason why the two specimens show brittle fractures is that the brazing temperature is not high enough, so that the elements of the brazing filler metal have not been completely diffused, resulting in poor mechanical properties of the joint. Figure 14c–e show the shear fracture morphologies of 3# (900 °C/20 min), 4# (920 °C/20 min) and 5# (940 °C/20 min). It can be clearly observed from the figures that there are a substantial number of shear dimples distributed. As the brazing temperature reaches 900 °C, the material changes from intergranular fractures and brittle fractures to transgranular fractures and ductile fractures. Furthermore, as the brazing temperature rises to 940 °C, the Widmanstätten structure continues to grow, and its growth direction is parallel. Due to the fact that the shear test will elongate the shear dimples, many fibrous parallel dimples can be observed in Figure 14e. With increasing brazing temperature, the parallel dimples become more pronounced.

**Figure 15:**
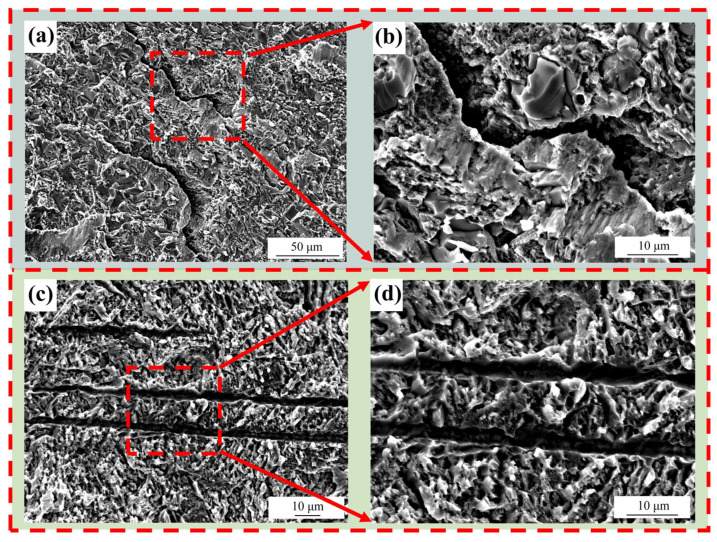
Fracture surface morphologies at different holding times when the brazing temperature is 900 °C: (**a**) fracture surface of the joint at 10 min, (**b**) the locally magnified fracture surface of Figure (**a**), (**c**) fracture surface of the joint at 30 min, (**d**) the locally magnified fracture surface of Figure (**c**).

Figure 15a–d, respectively, represent the fracture morphologies of the shear test for 6# (900 °C/10 min) and 7# (900 °C/30 min). For 6# (900 °C/10 min), due to the insufficient holding time, the brazing filler metal does not diffuse completely, resulting in poor joint performance. The fracture of the specimen has shear dimples of intergranular fracture, showing brittle fracture When the holding time is prolonged to 30 min, as in 7# (900 °C/30 min), there are parallel and transgranular shear dimples at the fracture. Meanwhile, the mechanical properties of the specimen joint are relatively good, indicating ductile fracture.

## 4. Conclusions

This paper studies the vacuum brazing process of TC4 alloy using Ti-Zr-Cu-Ni amorphous foil, and analyzes the influence of brazing temperature and holding time on the microstructure, element distribution, microhardness, and shear strength of the brazed joint. Furthermore, it clarifies the effect mechanisms of different brazing temperatures and holding times on the brazed joint. The conclusions are as follows:

(1) The increase in the brazing temperature leads to an intensification of the atomic thermal motion and a decrease in the diffusion activation energy, accelerating the diffusion rate of elements, thus widening the width of the joint interface. Additionally, with the increase in the brazing temperature, the Widmanstätten structure, influenced by the diffusion of titanium out of the base metal, continuously grows towards the center of the joint. When elements such as Zr, Cu, and Ni diffuse towards the base metal, (Ti, Zr)_2_(Cu, Ni) will be formed in the diffusion transition zone, making the joint more brittle and harder. Moreover, affected by the loss of Zr, Cu, and Ni elements and the relatively low diffusion rate of Ti elements, a Ti-rich zone is formed, which hinders the continuous diffusion of elements. At a brazing temperature of 900 °C, the brazing filler metal exhibits good diffusion, and the Widmanstätten structure does not yet become coarse, resulting in relatively good performance.
(2) Prolonging the holding time does not increase the width of the joint interface. Since extending the holding time does not accelerate the diffusion rate of Zr, Ni, and Cu elements, when the diffusion in the joint reaches a certain level, the diffusion of Ti elements towards the joint center will form a boundary layer of the Widmanstätten structure on the side of the base metal. In turn, the continuous diffusion of Zr, Ni, and Cu elements encounters more resistance. At a holding time of 30 min, the Widmanstätten structure continuously grows from the side of the base metal and extends to the central area of the joint, and the (Ti, Zr)_2_(Cu, Ni) keep growing, which is detrimental to the performance of the joint. Therefore, when the holding time is chosen to be 20 min, the comprehensive performance of the joint is the best.
(3) At a brazing temperature of 900 °C with a holding time extended to 20 min, the elements diffuse sufficiently, and (Ti, Zr)_2_(Cu, Ni) is evenly distributed in the transition zone. In addition, the Widmanstätten structure has not experienced coarsening, which makes the microhardness of the central area of the brazed joint reach the highest value, hitting 482.6 MPa.
(4) The shear strength is mainly affected by the width of the joint interface. The brazing temperature causes an increase in the width of the joint interface, thereby resulting in a higher shear strength. Increasing the brazing temperature to 940 °C raises the shear strength to 224 MPa. When the brazing temperature is held constant, the joint interface width remains unchanged, while prolonging the holding time promotes the development of the Widmanstätten structure. At a holding time of 20 min, elements diffuse sufficiently, leading to the highest shear strength of 137.6 MPa.
(5) This work investigated the effects of brazing temperature and holding time on the microstructure and properties of brazed joints, and guidance was provided for practical industrial applications. Since the size of sheets in this work is small, the holding time did not need to be kept too long. However, for large-scale parts in industrial practice, higher brazing temperatures or longer holding times are required compared to the small-sized samples in this work to ensure complete heat conduction to the joint center and full diffusion of atoms. Therefore, this study establishes the minimum thresholds for brazing temperature and holding time for industrial production.

## Figures and Tables

**Table 1:** Chemical composition of TC4 and Ti-Zr-Cu-Ni amorphous foil (wt%).

Materials	Ti	Al	V	Zr	Cu	Ni
TC4	Bal.	5.5~6.75	3.5			
Ti-Zr-Cu-Ni	Bal.			37.5	15	10

**Table 2:** Optimized brazing process parameters for TC4 with Ti-Zr-Cu-Ni.

Brazing Parameters for TC4 (Sample#)	Brazing Temperature (°C)	Holding Time (min)
1#	860	20
2#	880	20
3#	900	20
4#	920	20
5#	940	20
6#	900	10
7#	900	30

**Table 3:** Composition of points marked in Figure 7.

Points	Elements (at%)						Possible Phase
	Al	Ti	Cu	Zr	Ni	V	
1	7.0	35.5	16.8	24.3	14.7	1.7	(Ti, Zr)_2_(Cu, Ni)
2	10.3	42.6	9.4	28.1	9.7		eutectic phases
3	8.4	74.4	3.6	11.7		1.9	α-Ti (high Ti concentration)

**Table 4:** Composition of points marked in Figure 8.

Points	Elements (at%)						Possible Phase
	Al	Ti	Cu	Zr	Ni	V	
A1	2.0	38.6	14.7	30.5	14.3		(Ti, Zr)_2_(Cu, Ni)
A2	6.2	81.8	2.4	5.1	1.8	2.7	α-Ti (Widmanstätten structure)
A3	6.5	76.0	4.1	9.5	3.4	0.6	eutectic phases
B1	5.0	37.8	16.2	28.9	12.1		(Ti, Zr)_2_(Cu, Ni)
B2	6.2	73.0	4.7	11.1	3.1	2.0	eutectic phases
B3	3.3	76.6	3.7	12.2	1.3	3.0	eutectic phases
C1		37.5	11.6	35.1	15.8		(Ti, Zr)_2_(Cu, Ni)
C2	6.2	74.2	2.9	10.8	2.0	4.0	eutectic phases
C3	11.5	80.6	1.0	6.0	0.9		α-Ti (Widmanstätten structure)
C4		85.6	2.0	7.8	4.5		α-Ti
D1	13.1	36	11.6	27.5	10.0	1.9	(Ti, Zr)_2_(Cu, Ni)
D2	7.0	71.4	6.5	10.4	4.1		eutectic phases
D3	6.6	84.7		4.6	1.0	2.8	α-Ti

**Table 5:** Composition of points marked in Figure 11.

Points	Elements (at%)						Possible Phase
	Al	Ti	Cu	Zr	Ni	V	
E1	9.0	41.3	11.7	26.5	11.4		(Ti, Zr)_2_(Cu, Ni)
E2	6.6	79.5	3.2	9.0	1.7		α-Ti (Widmanstätten structure)
F1	10.7	78.1	2.5	6.1	1.0	1.5	α-Ti (Widmanstätten structure)
F2	1.6	78.9	3.4	6.3	2.7	7.0	eutectic phases

## Data Availability

The original contributions presented in this study are included in the article. Further inquiries can be directed to the corresponding author.
